# Hyperchloremia-associated acute chronic kidney injury: beware of confounders!

**DOI:** 10.1186/s13054-018-2291-7

**Published:** 2019-01-10

**Authors:** Patrick M. Honore, David De Bels, Luc Kugener, Sebastien Redant, Rachid Attou, Andrea Gallerani, Herbert D. Spapen

**Affiliations:** 1 ICU Department, Centre Hospitalier Universitaire Brugmann, Brugmann University Hospital, 4 Place Van Gehuchtenplein, 1020 Brussels, Belgium; 2 Ageing & Pathology Research Group, Vrije Universiteit Brussel, Brussels, Belgium

We read with great interest the recent paper by Oh et al. [1] investigating the association between perioperative hyperchloremia and postoperative acute kidney injury (AKI) in a large population of postsurgical patients admitted to the intensive care unit (ICU). One of the conclusions was that a substantial perioperative increase in serum chloride levels may reflect a higher risk of AKI in patients with moderate-to-severe chronic kidney disease (CKD) [1].

The authors exemplified some thoughtful limitations of the study. However, when looking carefully at the patient characteristics in the different cohorts, we came across several risk factors for AKI that were more present in hyperchloremic than in normochloremic subjects. The hyperchloremia group received significantly more radiocontrast (52.8 vs 28.4%; *P* < 0.001), non-steroidal anti-inflammatory drugs (NSAIDs; 40.2 vs 35.9%; *P* = 0.001), and diuretics, including mannitol and furosemide (69.3 vs 56.0%; *P* < 0.001). Also, patients who developed hyperchloremia had more severe shock as indicated by a higher need for inotropic and vasopressor support (87.2 vs 70.1%; *P* < 0.001) and more frequently underwent emergency surgery (17.5 vs 12.3%; *P* < 0.001). Contrast agents [2], NSAIDs [3], mannitol [4], and furosemide [5] are all associated with a potential deterioration of renal function in ICU patients and particularly in those with underlying CKD. In addition, emergency surgery and shock may markedly increase the incidence of AKI in the perioperative period. Taken together, the patient groups with and without hyperchloremia were not adequately balanced with regard to specific and independent AKI risk factors.

## Abbreviations

AKI: Acute kidney injury
CKD: Chronic kidney disease
ICU: Intensive care unit
NSAIDs: Non-steroidal anti-inflammatory drugs
